# Applications of Mesenchymal Stem Cells in Sinus Lift Augmentation as a Dental Implant Technology

**DOI:** 10.1155/2018/3080139

**Published:** 2018-04-16

**Authors:** Feridoun Parnia, Javad Yazdani, Solmaz Maleki Dizaj

**Affiliations:** ^1^Faculty of Dentistry, Tabriz University of Medical Sciences, Tabriz, Iran; ^2^Dental and Periodontal Research Center, Tabriz University of Medical Sciences, Tabriz, Iran

## Abstract

The potential application of stem cell biology in human dentistry is a new and emerging field of research. The objective of the current review was to study the efficiency of mesenchymal stem cells (MSCs) in sinus lift augmentation (SLA). A literature review was performed in PubMed Central using MeSH keywords such as sinus lift, MSCs, dental implants, and augmentation. The searches involved full-text papers written in English, published in the past 10 years (2007–2017). The review included *in vitro* and *in vivo* studies on the use of MSCs in SLA. Electronic searching provided 45 titles, and among them, 8 papers were chosen as suitable based on the inclusion requirements of this review. The reviewed studies have revealed the potential of MSCs in SLA. According to these papers, stem cell therapy combined with different biomaterials may considerably improve bone regeneration in previous steps of dental implantation and may veritably lead to efficient clinical usages in the recent future. However, the identification of an ideal source of stem cells as well as long-term studies is vital to assess the success rate of this technology. Further clinical trials are also needed to approve the potential of MSCs in SLA.

## 1. Introduction

Dental implants have been effectively applied for the replacement of dental elements since the earliest reports in the 1960s [1]. An appropriate success rate has been reported for dental implants; however, the success rates were revealed to be considerably low once the dental failures are measured according to persons who lost implants and not according to implants lost by the population [1–4]. Most of the implant-related techniques are evident as well as predictable; however, in some of the cases, there are problems related to the implant site that lead to failures in implant success [5, 6]. Considering the osseointegration and health of bone may have a wide influence on the long-term stability of implants [7–9]. Indeed, healthy gums and suitable bones are two necessary requirements to support the implants and to increase their success. Occasionally, both the low quality and unsuitable quantity of bone lead to an inappropriate implant site. Indeed, very thin or soft bone cannot support the implant, and thus, it will require a bone grafting process [7, 10].

The surgery process of adding dental bone into the upper jaw (in the molar and premolar areas) is recognized as sinus lift, sinus augmentation, or sinus lift augmentation. A sinus lift procedure is done once there is insufficient bone tallness in the upper jaw or once the sinuses are too close to the jaw for placement of dental implants. In this process, bone is added between the jaw and the maxillary sinuses (which are located on either side of the person's nose). In order to make an area for the bone, the sinus membrane has to be moved upward or lifted by a specialist (e.g., an oral surgeon or a periodontist) [4, 11–18]. Sinus lifting surgery can be open or closed. In an open sinus lifting process, a sufficiently large volume of bone tissue is created, while in a closed sinus lifting, the lack of a few millimeters of bone tissue is held [11, 15–17, 19].

Traditionally, the autogenous bone grafts taken from the same patient have been the standard for alveolar rebuilding. This is particularly important from the point of view of osteoconductivity as well as lack of immunogenic properties. However, there are some problems with autogenous bone graft treatment like infection or hematoma formation at the donor site as well as pain. Furthermore, a donor site with enough bone is not always accessible [20]. Bones taken from a different person (allograft bones) that are managed by a tissue bank or commercial supplier can also be applied. However, such a process also has problems such as unpredictable osteoinductivity, opposed host immune responses, and a deferred resorption, as well as a risk for prion and virus transmission [20, 21].

Recently, stem cell biology as an emerging field of research shows the ability to offer promising methods *in vitro* as well as *in vivo* in animal models with future applications in human dentistry. Experts are very optimistic that treatment of some severe conditions by stem cells can be made possible. Some reports have shown the success of stem cell use in dentistry such as regeneration of individual tissue types including bone, periodontal tissue, or someday even entire teeth.

Tissue engineering helps in the treatment of defective bone using stem cells with scaffolding materials to present bone-analogous constructs. Indeed, treatments based on stem cells paired with tissue-engineering technology can be successfully used for sinus lifting as well as bone regeneration. The efficiency of stem cells inside the host tissue is strictly related to the biomimetic properties of the scaffold. Most researchers have suggested the use of MSCs due to their good ability for osteogenic and chondrogenic differentiation. Numerous reports have confirmed that MSC-based treatment in combination with an osteoconductive scaffold or osteoinductive protein can be efficient in regenerating bone [22]. The type of scaffold is the main factor in this process that aids host cells to spread and multiply and simplifies their differentiation into bone-specific cells. Improvement of the scaffold's osteoconductivity is one of the tissue-engineering approaches. Bovine bone mineral (BBM), the most broadly used scaffold in sinus augmentation, has similar morphology and mineral composition with human cancellous bone [15].

The aim of this review was to study the efficiency of MSCs in sinus lift augmentation. The important question is, can such a technique veritably lead to efficient clinical usages in the recent future? Or, can stem cell therapy considerably improve bone regeneration prior to dental implant?

## 2. Method

### 2.1. Search Strategy

**Figure 1:**
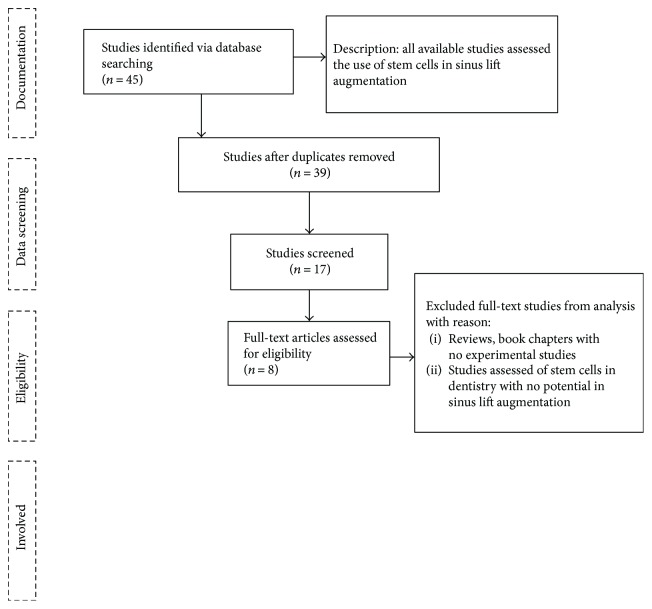
Searching strategy for the review.

A literature review was performed in PubMed Central using MeSH keywords such as sinus lift, stem cell, dental implants, and augmentation.  Figure 1 shows the searching strategy for the review.

### 2.2. Search Criteria

The search included *in vitro* and *in vivo* studies on the use of MSCs in sinus lift augmentation.

The study was limited to full-text papers written in English, published in the past 10 years (2007–2017).

Abstracts, reviews, books, or book chapters with no experimental data were excluded from the analysis.

Studies without the explicit involvement of the use of MSCs in sinus lift augmentation were excluded from further investigation.

### 2.3. Data-Screening Process

Data screening was done in two stages. The first stage included the use of EndNote 7 software for removing the duplicates, and the second stage was completed by reading the abstracts of the papers.

### 2.4. Search Results

Electronic searching provided 45 titles, and among them, 8 papers were chosen for presentation in the current review based on the screening process.

## 3. The Role of Stem Cells in Sinus Lift Augmentation

According to investigators, cell therapy combined with the use of bioactive materials may expressively improve bone regeneration prior to dental implant. To this aim, a perfect and proper cell source of progenitor cells is needed [23].

Stem cells are defined as undifferentiated cells with the ability to differentiate into specialized cells. The chief stem cells with the most applications are embryonic stem cells (ESCs) and adult stem cells, also known as pluripotent MSCs. MSCs are multipotent cells that can differentiate into a diversity of cells like osteoblasts. These types of cells have been recognized in living tissue as well as in tissue culture [14, 16, 20, 24, 25]. Among all probable options of stem cell sources, adult stem cells show some advantages over ESCs, umbilical cord stem cells, and amniotic fluid stem cells for regeneration of bone. This type of stem cells is chronologically similar to the target dental, oral, and craniofacial structures compared to other stem cells. Adult stem cells are also not subjected to the ethical controversy associated with ESCs [26].

According to the literature, treatments based on stem cells together with tissue-engineering technology can be efficiently used in maxillary sinus lifting. Reports have revealed that using bone marrow-derived mesenchymal stem cells (BM-MSCs) is a new strategy for maxillary sinus floor elevation. Indeed, bone tissue engineering based on stem cells has been known as a novel approach for maxillary sinus floor elevation. Based on scientific literature, bone formation may improve after simultaneous dental implant placements.

Zhou et al. tested osteoblast differentiation of BM-MSCs *in vitro*. Their results showed enhanced differentiation outcomes and greater new bone formation during maxillary sinus floor elevation under examination conditions. They tested the differentiation of BM-MSCs into osteoblasts under cyclic compressive pressure. Based on the obtained results, differentiation of BM-MSCs into osteoblasts was meaningfully improved under cyclic compressive pressure during sinus floor augmentation. According to the author's discussion, the pressured complex of BM-MSCs and Bio-Oss helped new bone formation and maturation in the rabbit maxillary sinus due to osteoconductive properties of Bio-Oss. In addition, BM-MSCs with the ability to regenerate a varied range of tissues such as bone and cartilage improved bone formation during maxillary sinus floor augmentation. They stated that after removal of pressure, the mRNA expressions of related genes continue at high levels and impact cell differentiation. Based on some evidence presented by investigators, cells interact with each other through integrins that lead to the production of intercellular adhesion and then alteration in the cytoskeleton that can change bone formation [15].

In a report by Kaigler, they showed that renovation of bone deficiencies of the maxillary sinus using stem cells can present potent views to improve treatment predictability for patient care. The authors assessed this examination using autologous cells that were enriched with CD90+ stem cells and CD14+ monocytes on thirty patients. Their radiographic tests exhibited no changes in the absolute bone volume obtained between selected groups. Then again, a higher volume of bone was observed in cases that received stem cells. Their results exhibited that stem cell therapy using enriched CD90+ populations is safe for maxillary sinus floor reconstruction, and they proposed its potential to accelerate and improve tissue-engineered bone quality in other craniofacial bone defects and deficiencies [16].

Yu et al. reported the capacity of bone derived from different sources for canine maxillary sinus augmentation in 6 beagles with 3 graft types including Bio-Oss granules alone (group A), a complex of osteoblasts derived from bone marrow MSCs (BM-MSCs) and Bio-Oss (group B), and a complex of osteoblasts derived from periodontal ligament stem cells (PDLSCs) and Bio-Oss (group C). They used conventional methods to assess new bone deposition as well as remodeling in the augmented part after 12 weeks. Their investigations showed a higher osteogenic capacity for groups B and C than for group A. They suggested that their tissue-engineered bone complexes might be an optimal selection for augmentation of the maxillary sinus in edentulous patients and due to seeding of PDLSCs or BM-MSCs onto Bio-Oss can encourage formation of new bone as well as mineralization. It also maintains the maximum augmented maxillary sinus [13].

Adult sheep are the most utilized models in dentistry. Valbonetti et al. have presented a report around a study on sheep sinus, using cone beam computed tomography (CBCT) to compare the sheep and human sinus morphological parameters, to standardize this model for using in the sinus augmentation trials. They tested 6 adult female sheep to determine the dimensions of the ovine maxillary sinus using CBCT. Their results showed that human and sheep maxillary sinus have anatomical differences that must be taken into account in experimental trials. Therefore, the standardization of the method should be done to decrease the error on the main parameters. They concluded that the sheep model could be appropriate for examining surgical trials of sinus augmentation in humans [27].

In a research work, Ardjomandi et al. investigated the usefulness of MSCs for sinus augmentation on 16 sheep models. Their results showed that bovine bone mineral (BBM) and MSCs in a mixture with fibrin adhesive were appropriate for new bone formation. They concluded that bone marrow aspirate concentrate (BMAC) can be suitable for human MSCs. However, it should be optimized for fitting with other cell features when it is applied to other models [24].

Oshima et al. reported the potency of novel gabapentin-lactam (GBP-L) in enhancing new bone formation in 10 adult sheep. They placed bovine bone mineral (BBM) and MSCs combined with novel GBP-L for each test sheep. BBM with similar physical properties (morphology and mineral composition) to human cancellous bone has been used to enhance the osteoconductivity of scaffolds. Then, they tested new bone formation using histomorphometry after 8 and 16 weeks. Their results based on a colony-forming unit and differentiation assay showed the osteogenic potency of the MSCs. Furthermore, the histologic test exhibited new bone formation in tight contact with the selected bone in both groups. The results also revealed that GBP-L caused to no alteration in the multipotency of the MSCs or loss of new bone forming. According to their suggestion, bone formation is initiated from the residual alveolar crest and along the implant. They stated that the elected mode of GBP-L application did not encourage the rapid forming of more fresh bone, and other forms of applications, such as slow release or systemic administration, might clarify the controversial *in vitro* findings [25].

Ricker et al. assessed and reported an outstanding implant and the clinical performance of this implant located in the posterior maxilla in 12 edentulous patients for 1 year. They treated one side with bovine bone mineral seeded with an iliac crest bone marrow enriched in MSCs as the test group and the other side with bovine bone mineral mixed with autogenous bone as the control side, which was done randomly. Their results showed no implant misplacing after functional loading, some implant failures in the test group, and no implants failing on the controls during osseointegration. Their results also exhibited no clinical alterations concerning soft tissue parameters or peri-implant bone loss after one year. Their test exhibited comparable 1-year postfunctional loading outcomes for the two methods for maxillary sinus floor elevation. However, the implant survival rate as a primary result, showed to be lower in the test group (91% compared to 100% in control group) [17].

Reports showed that adipose tissue-derived stem cells (ADSCs) and bone marrow stem cells (BMSCs) are the most important types of cells for bone renewal. However, in the case of the osteogenic potential of BMSCs, some reports resulted in differing assumptions. Zhang et al. tested and compared the potential for bone formation of ADSCs and BMSCs *in vivo*. Their results showed a better proliferative capacity together with larger osteogenic differentiation at the mRNA as well as protein levels for BMSCs. Their results also confirmed that in the presence of GFP-labeled cells on calcium phosphate cement scaffolds, only BMSCs formed new bone after subcutaneous implantation into nude mice. In addition, sequential fluorescence labeling leads to faster and higher bone regeneration for BMSCs through the examination time. According to the authors, apparent mineralization was also detected after implantation in the ADSC group. The authors introduced BMSCs as a more helpful rapid bone regeneration agent than ADSCs for sinus augmentation with simultaneous implant placement [14]. The studies related to stem cell therapy in sinus lift augmentation are summarized in Table 1.

Our literature review exhibited that MSCs are not the only type of stem cells capable of increasing the repair and regeneration of bone. In recent years, amniotic epithelial cells (AECs) have also been examined as a probable source of stem/progenitor cells for therapeutic aims [23]. Barboni et al. evaluated the bone regenerative property of the AECs as an emerging source of progenitor cells. The cells were loaded on a bone substitute comprised of calcium phosphate using a direct rapid prototyping (rPT) method in six adult sheep. Based on their reports, the scaffold integration was influenced by allotransplanted AECs. Their results also showed that sinus explants derived from sheep grafted with ovine amniotic stem cell- (oAEC-) engineered scaffolds exhibited a diminished fibrotic reaction and a restricted inflammatory result as well as an improved procedure of angiogenesis. Furthermore, oAECs significantly stimulated osteogenesis via increasing bone deposition or making more extensive the foci of bone nucleation. oAECs also showed direct participation in bone deposition that was related to the entrapped oAECs in the deposited osteoid matrix and through the ability of oAECs to switch on the expression of osteocalcin as a bone-related protein, when transplanted into host tissues [11]. In another work, Berardinelli et al. tested the role of a scaffold based on magnesium-enriched hydroxyapatite (MgHA)/collagen-based which was engineered with ovine amniotic fluid mesenchymal cells (oAFMCs) in a bone regeneration procedure on sinus augmentation of eight adult sheep. Based on their results, applying MSCs enhanced bone deposition and also initiated a faster angiogenic reaction. They proposed that the osteoinductive effect of a biomimetic commercial scaffold can expressively improve using oAFMCs. They concluded that the amniotic fluid mesenchymal cell (AFMC) can be recognized as a new and available source of MSCs to improve stem cell-related treatment for maxillofacial surgery [12].

Nauth and Schemitsch have also reported on the use of a new stem cell type for enhancing bone formation, named endothelial progenitor cells (EPCs). This type of cells, with a progenitor cell population of hematopoietic origin, is able to participate in angiogenesis [22].

## 4. Conclusion

There are some research reports related to the regeneration of individual tissue types, such as bone, periodontal tissue, or someday even entire teeth with stem cells. The literature showed that stem cell therapy combined with different biomaterials may considerably improve bone regeneration in previous steps of dental implantation and may veritably lead to efficient clinical usages in the recent future. However, the identification of an ideal source of stem cells as well as long-term studies is vital to assess its success rate. Furthermore, lacking sufficient knowledge about the cell population used as part of a stem cell therapy makes it problematic to recognize the mechanisms underlying the study results.

## Figures and Tables

**Table 1:** The research works related to stem cell therapy in sinus lift augmentation.

Aim of the research work	Type of experiment	Main finding(s)	Reference
Using BM-MSCs for maxillary sinus floor elevation and bone formation	*In vitro*	The differentiation of BM-MSCs into osteoblasts was significantly increased, and also, formation of new bone was enhanced after implantation of BM-MSCs during maxillary sinus floor elevation.	[15]
The usefulness of MSCs and the BMAC with BBM for sinus augmentation	*In vivo* (on 16 sheep)	New bone formation due to BBM and MSC application	[24]
Renewal of maxillary sinus deficiencies the using stem cells	On 30 human participants	Higher density of the engineered bone in patients that received stem cells Enriched CD90+ populations are harmless for maxillary sinus floor rebuilding, and they can also be used to accelerate and improve tissue-engineered bone quality.	[16]
Testing the potential of different stem cell sources about maxillary sinus augmentation	*In vivo* (6 beagles)	The tissue-engineered bone complexes might be a worthy choice for augmentation of the maxillary sinus in edentulous patients because seeding of PDLSCs or BM-MSCs onto Bio-Oss can encourage bone formation and mineralization.	[13]
Gabapentin-lactam (GBP-L) in enhancing new bone formation	*In vivo* (10 adult sheep)	The osteogenic potency of the MSCs and new bone formation in tight contact with the original bone	[25]
Sinus lifting using bovine bone mineral and autogenous bone marrow concentrate or autogenous bone	In 12 edentulous patients	No implants failed on the control side during osseointegration. No clinically related alterations concerning soft tissue parameters or peri-implant bone loss after one year	[17]
The bone formation capacity of ADSCs and BMSCs	In a canine sinus floor augmentation model	More proliferative ability as well as larger osteogenic differentiation potential for BMSCs Promoted fast and larger bone regeneration for BMSCs	[14]
The bone regenerative property of the amniotic epithelial cells (AECs) (an emerging source of progenitor cells) loaded on calcium phosphate	*In vivo* (in six adult sheep)	Allotransplanted AECs influenced positively on scaffold integration and bone deposition A decreased fibrotic reaction and a restricted inflammatory response as well as an improved angiogenesis Direct participation in bone deposition related to oAEC entrapment within osteoid matrix	[11]

## Abbreviations

SLA: Sinus lift augmentation
MSCs: Mesenchymal stem cells
ESCs: Embryonic stem cells
BM-MSCs: Marrow-derived mesenchymal stem cells
BBM: Bovine bone mineral
BMAC: Bone marrow aspirate concentrate
GBP-L: Gabapentin-lactam
ADSCs: Adipose tissue-derived stem cells
BMSCs: Bone marrow stem cells
AECs: Amniotic epithelial cells
oAECs: Ovine amniotic stem cells
MgHA: Magnesium-enriched hydroxyapatite
oAFMCs: Ovine amniotic fluid mesenchymal cells
AFMCs: Amniotic fluid mesenchymal cells.
